# From Immunobiology to Clinical Application: Tumor-Infiltrating Lymphocytes in Melanoma

**DOI:** 10.3390/jpm16030147

**Published:** 2026-03-03

**Authors:** Mislav Mokos, Mirna Šitum

**Affiliations:** 1 Department of Dermatology and Venereology, Sestre Milosrdnice University Hospital Center, 10000 Zagreb, Croatia; mirna.situm@kbcsm.hr; 2 School of Dental Medicine, University of Zagreb, 10000 Zagreb, Croatia; 3 Croatian Academy of Sciences and Arts, 10000 Zagreb, Croatia

**Keywords:** tumor-infiltrating lymphocytes, melanoma, adoptive cell therapy, immunotherapy, immune checkpoint inhibitors, tumor microenvironment, biomarkers, personalized medicine, lifileucel

## Abstract

**Background:** Tumor-infiltrating lymphocytes (TILs) play a key role in the immune response against melanoma. They act as both markers of an active tumor environment and as treatments in adoptive cell therapy. This narrative review covers what is currently known about TIL biology, their prognostic and predictive value, and the use of TIL-based adoptive cell therapy (TIL-ACT) in advanced melanoma. **Methods:** We searched PubMed/MEDLINE, Web of Science and clinicaltrials.gov through January 2026 using terms related to melanoma, TILs, adoptive cell therapy, immune checkpoint inhibitors, neoantigens, T-cell receptor clonality, and spatial transcriptomics. We included original research, major clinical trials, translational studies and key reviews. **Results:** Melanoma often has many neoantigens, which leads to a high number of tumor-resident TILs. These TILs, their arrangement, and their interactions with myeloid cells influence how well they fight tumors. Features of TILs seen under the microscope and through other tests can help predict patient outcomes, even before treatment. Studies show that TIL-ACT leads to objective responses in about 30–50% of patients whose melanoma did not respond to immune checkpoint inhibitors. Some patients achieve lasting complete remissions, though the treatment can cause significant, mostly short-term side effects from lymphodepletion and interleukin-2. New research points to factors related to the patient, tumor, and TIL product that affect treatment success, supporting the use of biomarkers and combination strategies. **Conclusions:** TIL-based adoptive cell therapy is now a promising, personalized treatment for advanced melanoma after anti-PD-1 therapy has failed. Future studies should focus on identifying reliable biomarkers, improving TIL products, combining therapies to change the tumor environment, and making manufacturing more efficient to ensure more patients can safely access TIL therapy.

## 1. Introduction

Cutaneous melanoma is an immunogenic cancer that harbors many ultraviolet (UV)-induced mutations that generate neoantigens recognized by T cells [1,2,3]. Immune checkpoint inhibitors (ICIs) that target CTLA-4, PD-1, and LAG-3 have shown that T-cell-mediated immunity is crucial for controlling melanoma [1,2,3,4]. However, many patients still face primary resistance or relapse after initial responses, and there are few effective treatments for those whose disease does not respond to ICIs.

Tumor-infiltrating lymphocytes (TILs) are T cells that develop naturally within the tumor environment. Earlier studies found that a strong lymphocytic infiltrate in primary melanoma is associated with better outcomes [5,6]. Later, researchers showed that expanding patient’s own TILs outside the body and reinfusing them after lymphodepletion can lead to real and lasting responses in metastatic melanoma [7,8]. This method, which uses each patient’s own tumor-derived T cells, is an early form of personalized cellular immunotherapy.

Interest in TIL-based adoptive cell therapy (TIL-ACT) has grown as more patients experience immunotherapy failure. Multiple studies have shown that TIL-ACT can help about 30–50% of patients with advanced melanoma that does not respond to anti-PD-1 therapy [9,10,11]. In a key phase II trial, lifileucel, a cryopreserved TIL product, led to a 36% response rate in patients who had already received numerous treatments, with some responses lasting over 18 months [11]. New phase III results showing TIL-ACT’s superiority over ipilimumab have made TIL therapy an important therapeutic model for patients whose disease is resistant to ICIs [12].

At the same time, new techniques like single-cell transcriptomics, spatial profiling, and T-cell receptor sequencing have shown that TIL biology depends on neoantigen specificity, T-cell differentiation, interactions with myeloid cells, and tumor resistance pathways [13,14]. These findings suggest that TILs may serve as biomarkers of the tumor immune microenvironment and as therapeutic agents in adoptive cell therapy.

Several systematic reviews and meta-analyses have summarized the clinical activity and toxicity profile of TIL-based adoptive cell therapy in melanoma and other solid tumors [15,16]. However, these quantitative syntheses are primarily designed to estimate response rates and safety outcomes and do not fully integrate rapidly evolving mechanistic data, including single-cell and spatial profiling, with clinically actionable questions such as which patients are most likely to benefit, which tumor and product features drive resistance or response, and how TIL therapy can be rationally optimized and positioned in current treatment algorithms. This narrative review was designed to address this translational gap by linking TIL immunobiology and tumor microenvironment context to biomarker interpretation, therapeutic resistance, and practical clinical implementation.

This narrative review synthesizes current knowledge on TILs in melanoma, focusing on advanced cutaneous melanoma and human clinical data, and it specifically bridges clinical evidence with emerging mechanistic and spatial insights to address biomarker guided application and resistance in the modern immune checkpoint inhibitor era. It underlines the translational potential of TIL therapy, moving from immunobiology to clinical application. We cover what affects TIL function, their predictive and prognostic value, the basics and results of TIL-ACT, and new ways to personalize and improve TIL-based immunotherapy and fit TIL therapy into current melanoma treatment algorithms.

## 2. Materials and Methods

This review brings together current evidence on TILs in melanoma, covering their immunobiology, potential as biomarkers, and use in adoptive TIL therapy. We searched PubMed/MEDLINE, Web of Science, and clinicaltrials.gov up to January 2026 using the following terms: “melanoma,” “tumor-infiltrating lymphocytes,” “TIL therapy,” “adoptive cell therapy,” “immune checkpoint inhibitors,” “neoantigens,” “TCR clonality,” and “spatial transcriptomics.”

We mainly included studies on adult patients with unresectable or metastatic cutaneous melanoma. Original research studies and review articles were screened, giving priority to important mechanistic studies, translational multi-omics analyses, prospective clinical trials of TIL-ACT (phases I-III), and consensus recommendations on TIL manufacturing and clinical use. Preclinical studies performed on mice or in vitro studies were included only if they directly informed on human TIL biology or clinical translation. Studies on non-melanoma tumors, non-peer-reviewed reports, and publications in languages other than English were excluded. We also reviewed the reference lists of key papers to identify additional primary sources.

This review was carried out as a narrative synthesis rather than a systematic review. Thus, we did not follow a PRISMA-style study selection process with formal record counts, nor did we conduct a risk-of-bias assessment. We summarize the findings qualitatively, focusing on evidence that is consistent across biological, translational, and clinical studies. This approach may favor well-documented trials and studies from high-resource centers, so the review should be read with this in mind.

## 3. Immunobiology of TILs in Melanoma

### 3.1. Melanoma Neoantigens and Tumor Antigenicity

Melanoma is highly immunogenic due to its large number of UV-induced somatic mutations, which create many neoantigens that T cells can recognize [17,18]. Neoantigen-specific CD8^+^ and CD4^+^ T cells are often found in TILs and usually make up most of the TCR repertoire in the tumor. These T cells target both unique neoantigens and common melanoma antigens like MART-1, gp100, and NY-ESO-1 [8,19,20,21].

Even though melanoma is highly antigenic, tumors can evade detection by interfering with antigen presentation. Loss of HLA class I, mutations in β2-microglobulin, and reduced expression of antigen-processing components all limit how well peptides are displayed. Problems with interferon-gamma (IFN-γ) signaling, especially mutations in Janus kinase (JAK) 1 or 2, stop the upregulation of antigen-presentation genes and make tumors resistant to T-cell killing and checkpoint blockade [22,23,24]. These resistance mechanisms also have practical implications for TIL product generation. When tumors acquire loss of HLA class I or β2-microglobulin, effective recognition by HLA class I restricted CD8 T cells can be reduced, which supports a manufacturing approach that prioritizes direct tumor-reactivity screening across multiple tumor fragments and aims to preserve a broad repertoire of tumor-reactive clonotypes rather than relying on a narrow set of dominant specificities [25,26]. Disruption of IFN-γ signaling through JAK1 or JAK2 loss further limits IFN-γ-driven upregulation of antigen presentation machinery, which can contribute to resistance to T cell mediated killing and immune checkpoint blockade. In this context, emerging functional data suggest that tumor-specific cytotoxic CD4 TILs may help address selected immune-escape phenotypes, because CD4 TIL cytotoxicity can be maintained against β2-microglobulin deficient melanoma and can remain effective despite JAK1 loss when melanoma cells constitutively express MHC class II [25,26]. These observations provide a rationale for ensuring that manufacturing workflows do not inadvertently exclude potentially beneficial cytotoxic CD4 populations when MHC class II positive tumor targets are present, and they further reinforce the value of tumor-reactivity assays that confirm functional killing rather than relying on IFN-γ inducibility alone [25,26]. Antigenicity is also affected by intratumoral heterogeneity and immune editing. Changes in neoantigen expression and subclonal mutations affect which T-cell receptor (TCR) types remain. Even in tumors with many mutations, a large number of infiltrating T cells may actually target viral antigens instead of tumor neoantigens [27,28].

### 3.2. Composition of the TIL Compartment

The TIL compartment in melanoma is made up of different immune cells, including CD8^+^ cytotoxic T cells, CD4^+^ helper and regulatory T cells, γδ T cells, innate lymphoid cells, and B cells. CD8^+^ T cells are the main effectors and are often tumor-specific or react to neoantigens. These cells usually show PD-1, CD39, or TOX, which reflects ongoing exposure to antigens [13,29]. Among CD8^+^ TILs, there are cells that are newly activated and others that are exhausted. TCF1^+^ progenitor-exhausted cells are found near antigen-presenting areas and help maintain immune responses during immunotherapy [30,31].

CD4^+^ TILs include Th1, Th2, Th17, T follicular helper, and regulatory T-cell (Treg) types. Th1 cells help cytotoxic responses by producing IFN-γ and activating antigen-presenting cells. In contrast, FOXP3^+^ Tregs limit effector activity through CTLA-4, IL-10, and TGF-β, so the balance between CD8^+^ T cells and Tregs is important for the immune response against tumors [32,33,34,35]. It has been shown that plasma cells and B cells, particularly in tertiary lymphoid structures (TLSs), assist in presenting antigens and influence T-cell activity [36,37]. This cellular diversity explains why TIL function, persistence, and potential for adoptive therapy can vary.

### 3.3. Spatial Architecture of the Tumor Microenvironment

The location of the immune cells within melanoma has a major impact on how TILs function. Tumors are usually described as inflamed, immune-excluded, or immune-desert, depending on whether CD8^+^ T cells enter the tumor nests, remain in the surrounding tissue, or are largely absent [38,39]. Inflamed tumors have a dense CD8^+^ infiltrate, IFN-γ–induced chemokines such as CXCL9/10, and PD-L1 expression. In contrast, immune-excluded tumors harbor lymphocytes around the tumor, separated from tumor cells by barriers in the tissue or blood vessels, often associated with TGF-β or β-catenin signaling [40,41].

Pathologists have long recognized that the location of TILs within a tumor is of high relevance. When TILs are distributed throughout the tumor or completely surround it, patients tend to have a lower rate of sentinel node metastases and higher survival rates [5,6]. Furthermore, it has been shown that a high number of CD8^+^ cells at the tumor edge is a favorable prognostic marker in both primary and metastatic melanoma [42,43]. TLSs, which include B cells, dendritic cells and T follicular helper cells, are associated with greater CD8^+^ infiltration and better responses to ICIs, likely because they help present antigens and recruit new immune cells [36,37].

### 3.4. Functional States of TILs

TILs can exist in several functional states, influenced by ongoing antigen exposure and the tumor environment. Effector CD8^+^ T cells produce granzyme B and perforin to destroy tumor cells directly. However, under constant stimulation, many of these cells become exhausted, showing reduced cytokine production and increased levels of inhibitory receptors such as PD-1, TIM-3, LAG-3 and CD39 [29,44]. Among these exhausted cells, TCF1^+^ progenitor-exhausted CD8^+^ T cells still can proliferate and create more cytotoxic effectors. These cells are found more often in patients who respond to PD-1 blockade [13,30]. Tissue-resident memory (TRM) T cells, identified by CD69 and CD103, are common in melanoma and are linked to better outcomes and lasting local immune control [45,46,47,48,49].

When the ratio of CD8^+^ T cells to Tregs is low, immune activity is usually less effective [32,34,50,51,52]. Metabolic challenges like hypoxia, lack of nutrients, and high lactate levels also weaken TIL function by reducing glycolysis and mitochondrial health. Checkpoint signaling makes these problems worse and increases exhaustion [53,54,55,56,57]. Together, these functional states affect how well TILs can survive, respond to checkpoint blockade, and grow ex vivo for adoptive cell therapy.

### 3.5. Tumor-Resident Versus Blood-Borne T-Cell Clonotypes

TILs include both tumor-resident and blood-borne T-cell clonotypes, which have different specificities and roles in therapy. TCR sequencing studies reveal that the main clonotypes inside tumors are usually neoantigen-specific and are rarely found in the blood, suggesting they stay in the tumor and are selected by local antigens [20,29,58,59,60,61,62]. In contrast, circulating T cells are more likely to be bystander clonotypes that target viral antigens instead of tumor neoantigens [28,63,64].

During TIL manufacturing, tumor-resident clonotypes tend to expand, especially those with PD-1 or CD39 expression [29,58,62,65]. After infusion, these clones can persist in the body, return to tumor sites, and are linked to lasting clinical benefit [21,58,60,66]. Single-cell multi-omics studies show that patients who respond to TIL therapy receive products rich in exhausted but cytotoxic tumor-resident CD8^+^ clonotypes that grow in culture and stay detectable after treatment. In contrast, non-responders often get products mainly made up of circulating clonotypes that do not form strong populations within tumors [14,62,66,67]. The tumor TCR repertoire, especially the presence of tumor-resident, neoantigen-specific clonotypes, is essential for TIL product quality and the chance of a positive response.

### 3.6. Myeloid–T Cell Interactions and CD8^+^–Myeloid Networks

Myeloid cells play a key role in shaping TIL function. Conventional type 1 dendritic cells (cDC1) support antitumor immunity by presenting tumor antigens and producing chemokines like CXCL9/10, which attract CXCR3^+^ CD8^+^ T cells. When cDC1 are present, TIL infiltration is increased and the response to immunotherapy is higher [68,69,70,71,72,73]. In contrast, M2-like macrophages and myeloid-derived suppressor cells secrete IL-10, TGF-β, arginase-1 and inhibitory ligands, leading to suppression of T-cells. These cells are more common in immune-excluded or immune-desert tumors, which are linked to poor outcomes [74,75,76,77,78,79,80].

Spatial profiling shows that effective immune responses rely on organized interactions between T cells and myeloid cells. Patients who respond to adoptive TIL therapy have tumors with CD8^+^ T cell and myeloid cell networks that are rich in type I interferon (IFN) activity and antigen-presenting myeloid cells. These features help keep T cells active and allow infused TILs to rejoin the tumor environment. In contrast, non-responders lack these networks and have more suppressive or immature myeloid cells [14,81,82]. These results underscore just how much the antigen-presenting myeloid niches are important for both supporting TIL function and the success of natural immunity and TIL-based therapy.

### 3.7. Tumor-Intrinsic Immune Resistance Pathways

Melanoma cells use several internal mechanisms to limit TIL recruitment and function. For example, changes in antigen presentation (including loss of HLA class I, mutations in β2-microglobulin and reduced expression of antigen-processing genes) help the tumor escape detection by CD8^+^ T cells. Disruptions in IFN-γ signaling, including JAK1/2 mutations, also prevent upregulation of the antigen-presenting machinery and provide the tumor with resistance to T-cell killing and immune checkpoint inhibitors [22,23,24,83,84].

Oncogenic pathways also shape the immune environment. When Wnt/β-catenin signaling is active, Batf3-dependent dendritic cells are excluded, and CD8^+^ T-cell infiltration decreases. TGF-β activates fibroblasts, increases matrix buildup, and strengthens stromal barriers. VEGF leads to the formation of abnormal blood vessels and increases the number of suppressive myeloid cells. Continued mitogen-activated protein kinase (MAPK) activation reduces melanoma antigen expression and creates an immunosuppressive cytokine environment [40,41,85,86,87,88,89,90,91,92,93]. Studies have shown that non-responders to TILs often have higher levels of TGF-β, VEGF, Wnt/β-catenin, and MAPK signaling, along with lower IFN and antigen-presenting cell activity. These features are linked to immune exclusion, weaker myeloid activation and less T-cell activity within the tumor [14,94,95,96,97].

## 4. TILs as Prognostic and Predictive Biomarkers for Personalized Management

### 4.1. Historical and Modern TIL Scoring Systems

Histopathology first identified TILs as a prognostic feature in melanoma (Table 1). The traditional brisk/non-brisk/absent classification shows how many lymphocytes are present and where they are in the vertical growth phase. A brisk, diffuse, or circumferential infiltrate is associated with a lower rate of sentinel lymph node involvement and higher melanoma-specific survival [5,6]. Later, semi-quantitative systems measured overall intensity or looked separately at lymphocytes inside the tumor and around it. Using immunohistochemistry for CD3, CD8, and FOXP3 made quantification more objective. High CD8^+^ density, especially at the invasive margin, is consistently linked to better outcomes, while high FOXP3^+^ infiltration or a low CD8^+^/FOXP3^+^ ratio predicts a worse prognosis [42,98,99,100,101,102].

Digital pathology now makes it possible to automatically measure TIL density, spatial gradients, and the proximity of lymphocytes to tumor cells. Newer methods, based on the colorectal Immunoscore, combine CD8^+^ densities inside and around the tumor to improve prognostic accuracy, but there is still no standard scoring system for melanoma [42,100,103,104,105,106]. Gene-expression signatures that show T-cell inflammation, such as IFN-γ-related gene sets, complement histology and are linked to survival and response to immunotherapy [107,108,109]. Together, these approaches show how important lymphocytic infiltration is for prognosis and highlight the need for practical, standardized tools that measure both the amount and location of TILs.

**Table 1:** Histopathologic, immunohistochemical, and digital scoring approaches for TIL evaluation in melanoma.

Approach	Method	Typical Readout	Strengths	Limitations	References
Classic histopathology (H&E)	Brisk/non-brisk/absent TIL pattern in the vertical growth phase (distribution and density; “diffuse/circumferential” vs. focal vs. none)	Ordinal categorical score (3-level)	Fast; no extra cost; historically validated; broadly understood; correlates with SLN status and melanoma-specific survival	Subjective; interobserver variability; limited granularity; may under-capture spatial compartmentalization and heterogeneity/sampling issues	[5,6]
Modern histopathology (H&E, semi-quantitative)	Semi-quantitative schemes incorporating density + distribution, often considering intratumoral vs. peritumoral/invasive-margin localization	Ordinal or semi-quantitative scale; sometimes separate compartment scores	Adds spatial nuance beyond classic brisk scoring; still feasible on routine slides	Non-standardized definitions across studies; ROI selection bias; still observer-dependent	[42,98,100,101,102]
Numerical H&E scoring	Numeric scoring systems designed to outperform 3-tier categorical scoring	Continuous or multi-level numeric score	Better risk stratification potential vs. brisk/non-brisk/absent; preserves more information	Manual scoring burden; training/standardization needed; adoption remains variable	[104]
Single-marker IHC (manual/semi-quantitative)	IHC quantification of CD3 and/or CD8, with emphasis on invasive margin and/or intratumoral compartments	Density (cells/mm^2^) or counts/HPF; % positive; high vs. low by cutoff	More objective subset-specific assessment than H&E; mechanistically interpretable; invasive-margin CD8 can be strongly prognostic	Antibody/platform variability; ROI selection (hotspots vs. whole section); inconsistent cutoffs across studies; tissue/scanner effects	[42,98,100,101,102]
Regulatory T-cell IHC/ratio metrics	IHC for FOXP3 (Tregs) and balance metrics such as CD8/FOXP3 ratio	FOXP3 density; CD8/FOXP3 ratio; high vs. low categories	Captures suppressive component; may improve prognostic discrimination beyond CD8 alone	Ratio performance depends on ROI definition and cutoffs; FOXP3 is an imperfect proxy for suppressive function; spatial context often not fully integrated	[42,98,99,100,101,102]
Digital pathology (H&E or IHC)—automated quantification	Automated measurement of TIL density and spatial localization (intratumoral vs. stromal vs. invasive margin)	Continuous density maps; distance-to-tumor metrics; standardized compartment densities	Scalable and reproducible once validated; enables spatial features difficult to score manually	Requires QC and external validation; domain shift (scanner/stain/site); annotation burden; clinical deployment/regulatory hurdles	[42,100,103,104,105,106]
AI/deep learning on whole-slide images	Deep learning to quantify TILs and characterize spatial organization (neighborhoods/clusters/phenotypes)	Learned spatial features; immune distribution phenotypes; correlations with outcomes/molecular programs	Captures complex non-linear spatial patterns; whole-slide scalability	Interpretability; generalizability across cohorts; hidden confounding (tissue artifacts/stain); requires diverse training data	[105]
Composite Immunoscore-like digital IHC	Composite score combining densities in intratumoral + invasive margin regions (e.g., CD8 +/− CD3), conceptually aligned with Immunoscore framework	Composite category (e.g., low/intermediate/high) based on densities in defined regions	Often improves prognostic signal vs. single-region scoring; operationalizes immune contexture	In melanoma, not uniformly standardized; region definitions/thresholds vary; needs prospective validation	[103,106]
Transcriptomic immune signatures (adjunct to histology)	Bulk-expression immune signatures (incl. IFN-γ-related gene sets) complementing morphologic TIL assessment	Continuous signature score; inflamed vs. non-inflamed categories	Quantitative; integrates multiple immune pathways; may detect immune activation not obvious morphologically	Bulk mixing (tumor + stroma); limited spatial localization; platform standardization and cost/access issues	[107,108,109]

Abbreviations: HPF, high-power field; H&E, hematoxylin and eosin; IFN-γ, interferon gamma; IHC, immunohistochemistry; QC, quality control; ROI, region of interest; SLN, sentinel lymph node; TIL, tumor-infiltrating lymphocyte; CD3: Cluster of differentiation 3; CD8: Cluster of differentiation 8; FOXP3: Forkhead box P3.

### 4.2. Intratumoral Versus Peritumoral TILs

The effect of TILs on prognosis depends on where they are found in relation to tumor cells. Intratumoral TILs, which directly infiltrate melanoma nests, show that the immune system has reached the tumor. These TILs are linked to fewer sentinel node metastases and better survival in primary melanoma, as well as improved outcomes in metastatic cases treated with immunotherapy [6,42,101,110,111,112].

Peritumoral TILs, especially CD8^+^ cells at the edge of the tumor, offer additional important information. A high number of peritumoral CD8^+^ cells is linked to longer progression-free and overall survival (OS), and suggests that the tissue and blood vessels at the tumor border allow immune cells to enter [42,105,106]. On the other hand, when T cells stay only in the tissue around the tumor, the tumor often shows an immune-excluded pattern caused by TGF-β-driven changes in the matrix or Wnt/β-catenin signaling, and these tumors usually respond poorly to systemic immunotherapy [40,41,79,89,95]. New digital methods that measure intratumoral and peritumoral areas separately show that using both measurements together gives better results than using either alone. This highlights the value of assessing where TILs are located.

### 4.3. TIL Subsets and Survival Correlations

The types of TILs present are closely related to patient outcomes. When there are more CD8^+^ cells in or near tumors, patients usually have fewer lymph node metastases, longer periods without relapse, and better OS. This pattern often matches a T cell-inflamed, IFN-γ-driven profile [6,42,101,109,111,112]. In contrast, higher numbers of FOXP3^+^ regulatory T cells or a low CD8^+^/Treg ratio are linked to poorer outcomes, highlighting the importance of the balance between effector and regulatory cells [32,51,52,99,113].

B cells and TLSs are also considered positive factors. Melanomas that have CD20^+^ B cells or mature TLSs usually show more CD8^+^ cells and better survival, likely because of stronger local antigen presentation and immune activation [36,37,114,115,116]. Other cell types, such as PD-1^+^ or CD39^+^ CD8^+^ T cells, are tumor-reactive and chronically stimulated. Their effect on prognosis varies, but often matches the presence of neoantigen-specific clonotypes [13,28,29,60,62]. CD4^+^ Th1 cells generally support good outcomes, while Th2 and Th17 cells have more mixed effects depending on the situation [34,117,118,119,120,121]. In summary, the most reliable signs of better survival are high CD8^+^ infiltration, the presence of B cells and TLSs, and a high effector-to-Treg ratio.

### 4.4. TILs as Predictors of Response to Immune Checkpoint Inhibitors

The characteristics of TILs at baseline play a key role in how patients respond to ICIs. Those who respond to anti-PD-1 therapy usually have a T cell-inflamed tumor environment, with many CD8^+^ cells at the tumor edge, higher CXCL9/10 levels, and increased PD-L1 in tumor and myeloid cells. In contrast, non-responders often have immune-desert or immune-excluded tumors [39,80,111,122,123,124]. Gene signatures linked to IFN-γ-driven T cell inflammation are associated with better responses and survival during PD-1 and CTLA-4 blockade, and these signatures are often found in tumors rich in CD8^+^ cells [109,125,126,127,128].

Detailed studies have shown that certain TIL subsets are especially important. Patients who respond to PD-1 blockade often have more TCF1^+^ progenitor-exhausted CD8^+^ cells, which can still multiply and become cytotoxic effectors when checkpoints are blocked. They also have more PD-1^+^ CD39^+^ CD8^+^ T cells, which are expanded clones specific to new antigens [13,29,31,129,130,131,132]. The presence of B cells and TLSs adds further predictive value. Namely, tumors with mature TLSs, CD20^+^ B cells, and T follicular helper cells tend to respond better, likely because of improved local antigen presentation and coordination of immune responses [36,37,133,134,135,136,137]. However, some tumors with many TILs do not respond due to tumor-specific resistance mechanisms, such as defects in antigen presentation or IFN signaling. Sometimes, even tumors with few TILs respond, showing that ICI sensitivity depends on many factors [13,22,40,123,138,139,140].

### 4.5. TILs in the Neoadjuvant Setting

Neoadjuvant immunotherapy provides insight into how TILs influence early treatment response. Giving short courses of anti-PD-1 or combination ICIs before surgery quickly activates the immune system within tumors, making it possible to directly observe changes in the tumor microenvironment during treatment. In clinical trials, patients who respond well show clear increases in intratumoral CD8^+^ T cells, more tumor-reactive clonotypes, and large areas of T cell-driven tumor destruction. These changes are closely linked to longer relapse-free survival [110,141,142,143,144,145]. In contrast, non-responders usually have little change in TIL density, continued immune-excluded patterns, and limited signs of tumor cell killing.

Neoadjuvant studies show that responders have both an increase in existing tumor-resident clonotypes and the addition of new circulating clones in the tumor. This highlights the role of baseline tumor-resident T cells and how ICIs can change the TIL makeup in real time [146,147,148,149,150,151,152]. For personalized medicine, the TIL-based measures discussed here are moving from just predicting outcomes to helping guide risk assessment and treatment choices. High CD8^+^ cell density, T cell-inflamed gene profiles, and favorable spatial patterns like infiltration at the tumor edge and TLS can help identify patients with immune-permissive tumors who are more likely to benefit from immunotherapies, including TIL-ACT. On the other hand, low or excluded TIL levels may suggest the need for stronger or combined treatments to turn “cold” tumors into “hot” ones. As combined scores using histology, digital pathology, gene expression, and blood markers improve, they are expected to support personalized plans for monitoring, therapy sequencing, and referrals for TIL therapy or other cell-based treatments. Validating these integrated, TIL-focused biomarker models in future studies will be key to achieving the full potential of personalized melanoma treatment.

## 5. TIL-Based Adoptive Cell Therapy in Melanoma

### 5.1. Principles of TIL Therapy

Adoptive cell therapy with TIL-ACT is founded on expanding and reinfusing a patient’s own T cells that already recognize tumor antigens. TILs taken from melanoma tumors are rich in tumor-specific and neoantigen-reactive clonotypes, which are often missing or rare in the blood [20,58,61,62,153,154,155,156]. Activating and expanding these cells outside the body enables sufficient production for treatment, even in patients with weak immune systems.

Before infusion, patients receive non-myeloablative lymphodepletion with cyclophosphamide and fludarabine to remove regulatory and competing lymphocytes and boost homeostatic cytokines. This is followed by high-dose interleukin-2 (IL-2) to help the infused TILs survive and multiply [15,157,158,159,160,161]. One main benefit of TIL therapy is its broad antigen recognition. Namely, TILs can detect many patient-specific neoantigens and common melanoma antigens, which lowers the chance of tumors escaping by losing certain antigens [15,21,160,161,162,163]. Still, the treatment’s success depends on having enough high-quality tumor-resident clonotypes, their ability to grow and last, and a tumor environment that supports these active cells [58,60,66,164,165,166].

### 5.2. TIL Isolation, Expansion, and Reinfusion

**Figure 1:**
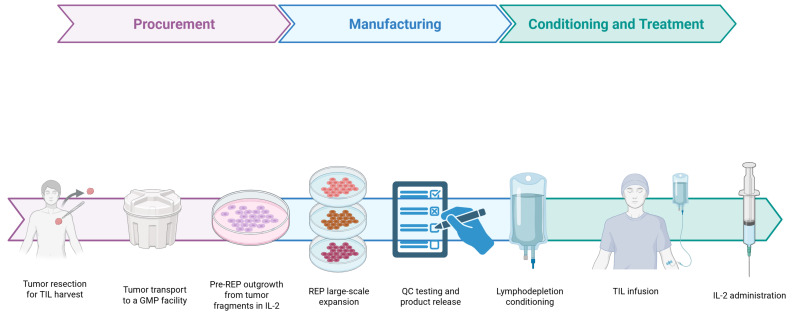
Clinical and manufacturing workflow of TIL-based adoptive cell therapy in melanoma. Tumor tissue is surgically resected and transported to a GMP facility where TILs are expanded from tumor fragments during pre-REP culture in IL-2, followed by large-scale REP using anti-CD3 stimulation, feeder cells, and IL-2. After quality control testing and product release (with optional cryopreservation/shipping depending on platform logistics), the patient receives lymphodepleting conditioning with cyclophosphamide and fludarabine, followed by TIL infusion and supportive IL-2 administration using standard or modified dosing schedules. Created in BioRender. Mokos, M. (2026) https://BioRender.com/byd1yt8 (accessed on 20 January 2026). Abbreviations: GMP, good manufacturing practice; IL-2, interleukin-2; QC, quality control; REP, rapid expansion protocol; TIL, tumor-infiltrating lymphocyte.

TIL therapy starts with surgically removing a metastatic melanoma lesion (Figure 1). The tissue is then fragmented and cultured in IL-2 to help lymphocytes grow from the tumor microenvironment [15,161,162]. In the pre-rapid expansion phase (pre-REP), these cultures increase the number of tumor-infiltrating T cells, including PD-1^+^ and CD39^+^ cells that have recognized tumor antigens in the body [29,167,168,169,170,171,172]. When enough cells are present, the cultures move to the rapid expansion phase (REP), where TILs are stimulated with anti-CD3, feeder cells, and high-dose IL-2 to produce billions of cells in about two weeks [158,162,173].

Centralized manufacturing is used to standardize important steps, lower the risk of production failures, and make it easier to use these therapies in more clinics while meeting regulatory standards [11,174].

### 5.3. Product Composition and Quality Attributes

The effectiveness of TIL products depends on their cellular composition. Successful products usually have more tumor-resident, neoantigen-specific CD8^+^ T cells that express PD-1, CD39, or TOX, which suggests they have already interacted with tumors and experienced ongoing antigen exposure [13,29,60,62,175]. TCR sequencing reveals that these tumor-resident cell types expand more during manufacturing and are the main cells that persist after infusion [58,60,66,176].

The differentiation state of TILs is also important. Batches with more TCF1^+^ progenitor-exhausted or central memory-like CD8^+^ T cells tend to grow better and last longer, while those with mostly terminally differentiated or senescent cells do not last as long [165,177,178]. Too many FOXP3^+^ regulatory T cells or non-tumor-specific bystander T cells can weaken the product. How TILs are made affects these qualities. Namely, REP leads to strong growth but often produces short-lived cells, while using different cytokines, changing activation strength, or selecting PD-1^+^ or CD39^+^ cells can help expand less differentiated, tumor-reactive cells [167,179,180,181,182]. Standardized products like lifileucel usually have a mix of cell types and are mostly made up of CD8^+^ effector-memory cells [11,174]. Setting clear quality standards, such as measuring TCR diversity, tumor reactivity, and markers that predict persistence, is still a key goal, since the makeup of the product is crucial to how well it works.

### 5.4. Comparison of Traditional and Centralized Manufacturing Platforms

Traditional TIL production began as a single-center process where on-site institutions performed tumor dissection, pre-REP expansion, REP, and product formulation [160,161,173,183,184,185]. This approach offered flexibility and close collaboration with clinical teams, but it needed specialized infrastructure, significant labor, and large tumor samples. Manufacturing usually took 3 to 6 weeks and often resulted in production failures.

Centralized or industrialized platforms were created to overcome these challenges. Facilities that make standardized products, such as lifileucel, use optimized, partly automated workflows with standardized culture conditions and validated quality controls. This shortens production time, reduces variability, and allows for cryopreservation of intermediate or final products [11,174,183,186,187]. These systems can process smaller or more varied tumor samples and help patients with limited resectable disease [11,187,188,189,190,191]. However, they offer less opportunity for customizing culture conditions or selective enrichment [157,192,193,194,195]. As traditional and centralized methods become more aligned, multicenter trials have become easier, paving the way for broader clinical use of TIL therapy [160,174,186,196,197].

### 5.5. Lymphodepletion and IL-2 Support

Non-myeloablative lymphodepleting chemotherapy is a standard part of TIL therapy. Cyclophosphamide and fludarabine are used together to lower the number of existing lymphocytes, reduce regulatory and competing T-cell populations, and boost homeostatic cytokines like IL-7 and IL-15. This approach helps the infused TILs engraft more effectively [158,159,160,161,198]. Early use of total body irradiation increased cytokine levels but also caused much higher toxicity, so it is now rarely used [165,186,199,200].

After TIL infusion, IL-2 is given to help T-cells grow and survive. Older protocols used high-dose IL-2 every 8 h until patients could no longer tolerate it, which often led to capillary leak and organ problems, but may have been important for long-lasting results in some cohorts [15,160,166,186,196,201,202]. Recent studies have tested lower, intermediate, or shorter IL-2 courses to reduce side effects, including options for older patients or patients with comorbidities [9,203,204,205]. In the C-144-01 study, a set cyclophosphamide–fludarabine regimen, a fixed TIL dose, and up to six high-dose IL-2 doses led to good responses in patients who had already received many treatments [11,174,206]. Researchers are also studying IL-2 variants and other γ-chain cytokines to improve TIL persistence and lower toxicity [201].

### 5.6. Clinical Implementation and Logistics

To successfully implement TIL therapy, teams need to work closely together across different specialties. Patients must have at least one metastatic lesion that can be removed and is large enough to create a viable TIL product. They also need to be healthy enough to handle surgery, lymphodepletion, and the side effects of IL-2 [160,186,197,207]. Quick assessment is important because waiting too long to collect the tumor can let the disease progress and make the patient ineligible.

The steps of TIL therapy need to be carefully timed with manufacturing, inpatient bed availability, and support services. Centralized manufacturing helps by using standard culture methods and quality checks, but it still requires careful coordination for shipping tumors, releasing products, and preparing patients at different hospitals [11,186,187,208,209]. Experts suggest that TIL therapy should first be offered at centers experienced with high-dose IL-2 and complex cancer care, with clear processes for choosing patients, planning surgery, preventing complications, providing transfusions, and monitoring after treatment [186,196,208]. As commercial TIL products become available, having efficient referral systems and standard protocols will be important so that eligible patients can get treatment quickly and safely at different locations [186,196,204,209].

## 6. Clinical Efficacy of TIL Therapy in Melanoma

### 6.1. Academic Single-Center Experiences

The first use of TIL therapy in metastatic melanoma was at single centers, mainly at the U.S. National Cancer Institute (NCI). In phase II studies, patients had their metastatic tumors removed, following which TILs expanded outside the body; received non-myeloablative cyclophosphamide and fludarabine for lymphodepletion; and were given high-dose IL-2. These studies showed overall response rates (ORRs) of about 50–70%, with complete responses (CRs) in 12–22% of patients. Many of these CRs lasted more than five years [158,160,200]. More intense regimens that included total body irradiation led to even higher response rates but caused more toxicity, so they are now rarely used [200].

These studies showed that TIL-ACT can lead to high response rates in patients who have already had several treatments, including high-dose IL-2 and chemotherapy. Some patients even achieved long-term, treatment-free survival, suggesting the therapy could be curative for a subset [160,200]. The benefit was linked to the number of TILs given and how well they reacted to tumors in the lab, highlighting the importance of the expanded cells [166,200]. Centers in Denmark and the Netherlands found similar results, with response rates around 40–50% and lasting responses and survival like those seen at the NCI. They also found that patients with lower tumor burden, normal lactate dehydrogenase (LDH), good performance status, and more CD8^+^ cells with strong tumor-specific activity in the product had better outcomes [9,166].

### 6.2. Multicenter Trials and Standardized TIL Products

Multicenter studies have proven the efficacy of TIL therapy in a wider range of patients, and this has led to the development of standardized commercial products (Table 2). In the phase II C-144-01 trial, lifileucel, a centrally made and cryopreserved TIL product, was tested in patients with unresectable or metastatic melanoma who had already received anti-PD-1/PD-L1 therapy and, when needed, BRAF/MEK inhibitors. The combined group had an ORR of about 36%, strong disease control, and a median duration of response that was not reached at the main analysis, with many patients still responding after one year [11]. The benefit was seen in patients with different baseline features, including those with high tumor burden and previous combination immunotherapy. Long-term follow-up showed a safety profile similar to what is expected with lymphodepletion and high-dose IL-2 [11].

Randomized studies add further support to these findings. In a phase III trial of patients with advanced melanoma who had progressed after anti-PD-1 therapy, TIL-ACT led to a much higher ORR and longer progression-free survival (PFS) than ipilimumab. There was also a positive trend toward better OS, even though some patients switched treatments [12]. Overall, these studies confirm that standardized TIL products are effective in checkpoint-refractory melanoma and support their approval and inclusion in treatment guidelines.

### 6.3. Subgroup Analyses and Special Melanoma Subtypes

Current data show that TIL therapy works across different molecular and clinical groups, although there is less evidence for these groups than for cutaneous melanoma overall. In the C-144-01 study, both BRAF-mutant and BRAF wild-type patients responded, even those who had already received BRAF/MEK inhibitors. This suggests that earlier targeted therapy does not rule out benefits from TIL-ACT [11]. Patients with high tumor burden and elevated LDH had lower response rates and shorter PFS, but some still experienced lasting responses [11].

Patients with brain metastases are especially high-risk. Early reports from single centers showed occasional responses in brain lesions, but many studies left out patients with active or unstable brain disease [9,160]. New real-world data suggest that TIL-ACT can be given safely to some patients with treated or stable brain metastases. However, controlling brain tumors remains difficult, and it is hard to know how much TILs help compared to local treatments [186,210].

Non-cutaneous types like acral and mucosal melanoma usually have fewer mutations and do not respond as well to ICIs. Still, small groups treated with TIL-ACT have shown meaningful results. In a Japanese pilot study of ICI-resistant melanoma, including acral and mucosal cases, one acral melanoma patient had a partial response and another had stable disease that lasted, even after heavy pretreatment and with lower expected immunogenicity [196,204,211,212]. Retrospective studies also support TIL-ACT as a backup option for patients with aggressive disease, as long as enough tissue can be collected and the patient is well enough for intensive treatment [9,16,166,199]. Larger studies are needed to better understand the benefits in these groups.

### 6.4. Durability of Response and Long-Term Outcomes

One key feature of TIL therapy for melanoma is its potential to provide long-lasting remission. In NCI trials, many patients who achieved a CR stayed disease-free for years. Most complete responders were still alive and free of recurrence after five to ten years, which suggests that the treatment may be curative for some patients [9,15,158,160,186,199,206,213]. Some patients also had durable partial responses or long periods of stable disease.

Mechanistic studies suggest these results are linked to the persistence of tumor-reactive T-cell clonotypes. Furthermore, long-term TCR sequencing and functional tests show that neoantigen-specific CD8^+^ T cells from TIL products can be found in the blood for years and still recognize the patient’s own tumor cells [21,58,186,214,215,216]. In patients who respond, these T-cell clonotypes often return to remaining tumor sites and show signs of ongoing immune activity [14,66]. In the C-144-01 study, the median duration of response was not reached at the main analysis, with many patients maintaining disease control for more than 18 to 24 months, even though they had already received several treatments and did not respond to checkpoint inhibitors. Moreover, long-term follow-up has not found any new late safety issues beyond what was seen with the initial therapy [11].

### 6.5. Comparison with Other Systemic Therapies and Treatment Sequencing

The main treatments for advanced melanoma are ICIs and, for patients with BRAF mutations, BRAF/MEK-targeted therapy. Combining anti-PD-1 with anti-CTLA-4 leads to ORRs of about 50–60% and long-lasting survival for some, but many patients do not respond or eventually relapse [2,217,218,219]. Using anti-PD-1 with anti-LAG-3 is another option that can be easier to tolerate, though resistance is still common [1,220]. In BRAF-mutant melanoma, BRAF/MEK inhibitors often work well at first, but resistance develops and PFS is usually short [3,221,222].

When anti-PD-1 therapy stops working, standard treatments like ipilimumab, ipilimumab plus nivolumab, or chemotherapy usually have response rates of only 10–15% and do not last long [223,224,225,226,227,228]. TIL-ACT is different because it uses tumor-resident T cells grown outside the body, instead of targeting more immune checkpoints. In a phase III trial, TIL-ACT led to higher response rates and longer PFS than ipilimumab in patients whose disease progressed after anti-PD-1 therapy, and OS was also better [12]. The lifileucel C-144-01 trial found similar results, with response rates higher than those seen with ICI rechallenge or chemotherapy in similar patients [11].

Combining or sequencing these treatments, including the use of ICIs to prime the tumor microenvironment before TIL-ACT or checkpoint inhibitors after TIL-ACT, has also been studied [186,196,208].

## 7. Safety and Toxicity of TIL Therapy

### 7.1. Safety Profile and Patient-Reported Burden

Most of the toxicity seen with TIL therapy comes from lymphodepleting chemotherapy and high-dose IL-2, not from the TIL product itself. Early NCI trials using cyclophosphamide and fludarabine for lymphodepletion often led to grade 3–4 cytopenias, frequent need for transfusions, febrile neutropenia, and opportunistic infections. Adding total body irradiation increased the risk of infections and mucosal complications, so it is now rarely used [158,160,161,194,200,229]. High-dose bolus IL-2 can cause capillary leak syndrome, leading to hypotension, tachycardia, fluid overload, and reversible organ dysfunction, along with gastrointestinal, general, and neurocognitive symptoms [160,166,202,230].

In contemporary programs, almost all patients experience severe blood-related side effects and a high rate of febrile neutropenia. IL-2 can also cause hypotension, hypoxia, and transient organ dysfunction, but these are usually manageable in experienced centers. Deaths related to treatment are rare and mostly linked to infections or organ failure [9,11,174,186,196,204,206,231]. Furthermore, patients often need a long hospital stay with significant symptoms like fatigue, stomach issues, fever, and unstable blood pressure [8,9,12,160,208]. Most patients who finish treatment return to their usual level of function within weeks or months, and those who respond well can have long periods without further treatment. However, recovery may take longer for patients with other health problems or lower fitness [9,11,185,186,204,206,208]. Compared to outpatient immunotherapies or targeted drugs, which tend to cause milder but longer-lasting side effects, TIL-ACT is a short but intense treatment that can strongly affect daily life for a limited time. Side effects directly caused by the TIL product, like autoimmune reactions or cytokine release-like syndromes, are rare and usually go away quickly [12,160,186,196]. Overall, TIL therapy causes significant but short-term side effects and a temporary drop in quality of life, requiring specialized hospital care. So far, long-term safety issues beyond those expected from chemotherapy have not been widely seen in follow-up.

### 7.2. Strategies to Mitigate Toxicity and Patient Selection

To reduce toxicity, efforts have focused on improving conditioning regimens, IL-2 dosing, and patient selection. NCI protocols found that adding total body irradiation to chemotherapy increased blood and infection-related side effects without a clear benefit [200]. Most contemporary regimens therefore employ cyclophosphamide–fludarabine-based lymphodepletion without irradiation, maintaining efficacy while lowering treatment-related morbidity [9,160]. The lifileucel program set a maximum of six high-dose IL-2 doses, showing that good response rates are possible with less cytokine exposure than older protocols [11].

Careful patient selection is key to reducing risk. Guidelines recommend TIL therapy only for patients with ECOG performance status of 0–1, good heart and lung function, healthy kidneys and liver, and enough social support for a demanding hospital stay [186]. Patients are usually excluded if they have uncontrolled brain metastases, serious infections, or organ problems that prevent lymphodepletion or IL-2 treatment. Before starting, patients undergo a detailed medical examination that allows current TIL programs to keep the therapy effective while lowering side effects and making sure the benefits outweigh the risks for the right patients.

An emerging strategy to reduce the toxicity and logistical burden of systemic IL 2 is to develop engineered TIL products that provide cytokine support without exogenous IL 2 [232]. OBX 115 is an autologous tumor derived TIL product engineered to express membrane bound interleukin 15 in a pharmacologically regulatable manner, which is intended to allow manufacturing and post infusion support without high dose IL 2. In this platform, membrane bound IL 15 expression is controlled using the small molecule acetazolamide, which enables outpatient redosing to support persistence and activity of transferred cells [232]. In the first in human clinical experience in immune checkpoint inhibitor resistant advanced melanoma, a per protocol efficacy cohort reported an objective response rate of 44.4% with complete responses in 22.2% and a disease control rate of 100%, with a reported progression free survival rate at 24 weeks of 75% [232]. Treatment was administered without IL 2, and early safety reports describe no dose limiting toxicities and no confirmed cytokine release syndrome, neurotoxicity, or capillary leak syndrome, with no intensive care unit care required. In the ongoing multicenter phase 1 and 2 study, preliminary results in patients treated at the recommended phase 2 dose have also shown encouraging activity, with an objective response rate of 67% and disease control in all treated patients at that dose level, supporting continued evaluation of IL 2 free engineered TIL approaches as a next step in reducing toxicity while preserving efficacy [232].

## 8. Towards Personalized Application of TIL Therapy

### 8.1. Patient-Related Determinants

Clinical outcomes with TIL therapy differ widely between patients because of factors like immune health, ability to tolerate treatment, and how well T-cell responses last (Table 3). Patients with ECOG performance status of 0–1, few other health problems, and good organ function are more likely to complete lymphodepletion and IL-2 and see positive results [9,11,160]. High baseline LDH, widespread disease, and large tumor burden are linked to worse outcomes, but some patients still benefit [11,166,174,233]. Age matters less than overall health. Namely, well-chosen older patients can benefit too, though frailty, heart risks, and lower marrow reserve can make intensive treatments harder to tolerate [160,185,186,208].

A patient’s immune health also affects the efficacy of TIL therapy. Previous treatments, especially several rounds of chemotherapy or long-term steroid use, can weaken lymphocytes and lower the quality of TIL products [15,165,166,199,213,234]. On the other hand, patients whose cancer has progressed after immune checkpoint inhibitors often still have many tumor-resident T cells that can be collected and expanded, making them good candidates for TIL-ACT [29,58,66,186,199]. Genetic and immune factors, like HLA type and inherited variants, may also play a role, but clear links have not yet been found in TIL-treated patients. These factors highlight the need to weigh risks and benefits for each patient when considering TIL therapy.

### 8.2. Tumor-Related Determinants

Tumor-intrinsic factors play a major role in how suitable and effective TIL therapy will be. Tumor mutational burden (TMB) and neoantigen load are important for determining antigenicity. Melanoma usually has a high TMB, which supports the presence of neoantigen-specific TILs. However, differences between patients and subtypes, like lower TMB in acral and mucosal melanoma, can change the diversity and strength of tumor-reactive responses [17,18,204,235]. The ability to present antigens is also crucial. Loss of HLA class I, mutations in β2-microglobulin, or defects in IFN-γ signaling can make tumors less visible to CD8^+^ T cells and may cause resistance to both ICIs and TIL-ACT [13,22,24,84,236].

The baseline immune environment of a tumor also helps predict TIL therapy outcomes. Tumors with an inflamed profile, high CD8^+^ T cell levels, and T cell-inflamed gene signatures are more likely to produce TILs rich in tumor-resident, neoantigen-reactive cells [29,58,111]. Studies show that existing networks of CD8^+^ T cells and myeloid cells, especially those with type I IFN-activated myeloid states, are common in patients who respond well to TIL therapy. In contrast, tumors with immune-excluded or immune-desert patterns often have less favorable TIL compositions and lower response rates [14,66,237,238,239]. As noted in Section 3.7, activation of the Wnt/β-catenin, TGF-β, VEGF, and MAPK pathways can block dendritic-cell recruitment, lower antigen presentation, and create stromal barriers. Tumors with these features are often less responsive to TIL-ACT in studies [14,40,84,204,240,241,242]. Tumor burden and lesion features are also important. Large, necrotic, or poorly vascularized metastases are harder to harvest TILs from and less accessible to T cells, while smaller, well-vascularized lesions are better sources for TIL generation [191,243].

### 8.3. TIL Product Characteristics as Biomarkers

The characteristics of the final TIL product offer valuable information for predicting patient outcomes. Early research found that a higher total TIL dose and stronger tumor reactivity in lab tests, measured by IFN-γ release or killing of the patient’s own tumor cells, are linked to better response rates and longer PFS [158,166]. Products with more CD8^+^ T cells and strong recognition of several tumor targets tend to perform best, while high levels of FOXP3^+^ regulatory T cells or bystander cells can reduce effectiveness [19,28,29,58,166,185,186,194,213]. Finding PD-1^+^ CD39^+^ CD8^+^ T cells suggests the product is rich in tumor-reactive cells that have been chronically stimulated, which is associated with better outcomes in both checkpoint and TIL-treated melanoma [29,58,60,154,175,233,242,244,245,246].

The way TILs are differentiated and their clonotype makeup also affect how well they work. Products with more less-differentiated, TCF1^+^ progenitor-exhausted, or central memory-like CD8^+^ cells tend to multiply better and last longer in the body. In contrast, products with mostly terminal effector or aging cells are linked to shorter-lasting responses [60,129,165,178,242,247,248]. TCR sequencing showed that a good therapeutic response was associated with products containing tumor-resident, neoantigen-specific clonotypes that were already present in the tumor and persisted after treatment [21,58,60,66,233,249]. Therefore, new biomarker strategies for predicting TIL therapy results include tumor reactivity, cell type, differentiation, and clonotype mix.

### 8.4. Spatial Biomarkers and Microenvironmental Context

Spatial organization of immune cells within the tumor microenvironment provides additional biomarkers for TIL therapy beyond bulk density and phenotype. Quantitative pathology studies show that high CD8^+^ T-cell density at the invasive margin, together with intratumoral infiltration, is associated with improved survival and reflects a permissive stromal and vascular architecture [42,43,100,250,251,252,253,254]. The presence of TLSs and B-cell-rich aggregates further indicates coordinated infiltration of CD8^+^ T cells, T follicular helper cells, and dendritic cells, and is associated with improved prognosis and higher response rates to ICIs [36,37,114,115,116,133,135,255]. TLSs likely serve as local hubs for antigen presentation, T-cell priming, and maintenance of tumor-reactive clones, thereby enhancing the quality of TILs that can be harvested and expanded.

Spatial multi-omics studies have identified more complex CD8^+^ T cell–myeloid cell networks as predictors of response to TIL therapy. In checkpoint-refractory melanoma treated with TIL-ACT, responders had baseline tumors enriched in intratumoral clusters where CD8^+^ T cells closely interacted with type I IFN-activated myeloid cells and tumor-intrinsic antigen-presentation and IFN pathways were upregulated, whereas non-responders lacked such networks despite similar overall TIL densities [14,66,67,176,256,257,258]. These spatial biomarkers emphasize that the microanatomical context in which TILs reside, encompassing invasive margin architecture, TLS formation, and CD8–myeloid networks, modulates the effectiveness of both endogenous and adoptively transferred T cells and should be incorporated into future biomarker strategies.

### 8.5. Integrating Biomarkers into Clinical Decision-Making

No single biomarker can reliably identify the best candidates for TIL therapy. However, research suggests that using a combination of clinical, tumor, and product-related factors is more effective. Patients who are in good health, have few other medical issues, and have controlled extracranial disease are most likely to handle treatment and benefit from it [160,185,186,196,197,208,249]. High LDH levels and a large tumor burden are negative factors, but they do not rule out a positive response, especially if other immune features are favorable [11,166,174,186,206,259,260]. Tumors with a T cell-inflamed profile, high CD8^+^ cell density, preserved antigen presentation, and intact IFN signaling, along with spatial markers like TLSs and CD8^+^ T cell–myeloid cell networks, are more likely to respond well to TIL-ACT [14,29,36,60,66,67,84,111,116].

Certain features of the TIL product, such as high CD8^+^ cell content, strong tumor reactivity in lab tests, more PD-1^+^ CD39^+^ tumor-reactive clonotypes, and a favorable mix of TCF1^+^ progenitor-exhausted or central memory-like cells, are becoming important markers of how well the therapy might work [16,29,58,158,165,166,178,213,244,261]. Right now, these factors are mostly used in a general way, not as strict rules for making decisions. Experts agree that TIL therapy is best for patients with tumors that can be removed and who are healthy enough for treatment, especially if they have not responded to anti-PD-1 treatments and their tumors still show signs of T-cell activity and are likely to provide a strong TIL product [16,196,197,208,209,262]. There is still a need for studies that confirm how well combined biomarker approaches work in practice.

**Table 3:** Candidate biomarkers of response and resistance to TIL therapy.

Biomarker Class	Candidate Determinant	How Assessed (Examples)	Association/Interpretation	Evidence Level in TIL Therapy	Clinical Feasibility	References
Patient dependent	Performance status/physiologic fitness (incl. comorbidities, organ function)	ECOG/PS; clinical assessment; labs/organ function; baseline cardiopulmonary reserve	Better tolerance of lymphodepletion + IL-2 and higher likelihood of benefit; poor fitness increases risk	Clinical association in TIL cohorts; used routinely in selection	Routine (standard clinical data)	[9,11,160,185,186,196,197,208,249]
Patient dependent	Baseline LDH and tumor burden/visceral disease extent	Serum LDH; imaging-based tumor burden; metastatic sites (visceral/brain)	Adverse prognostic factors; may still benefit when other favorable immune/product features present	Clinical association; mostly retrospective/observational; incorporated qualitatively in practice	Routine (standard clinical + imaging)	[11,166,174,186,206,233,259,260]
Patient dependent	Age and physiologic reserve (frailty, marrow reserve)	Chronologic age plus frailty/functional status; baseline hematologic reserve	Age alone less predictive than reserve; reduced reserve can limit tolerance of intensive regimens	Clinical association (selection/tolerability); not a standalone exclusion criterion	Routine (clinical assessment + CBC)	[160,185,186,208]
Patient dependent	Prior treatment intensity (multiple lines; cytotoxic chemotherapy exposure)	Treatment history; prior cytotoxic lines and cumulative burden	Heavily pretreated patients may have impaired immune competence and reduced TIL product quality	Clinical association; signals for product yield/quality are emerging	Routine (history); impact may require specialized correlates	[15,165,166,199,213,234]
Patient dependent	Checkpoint-refractory status/post-ICI progression	Prior anti-PD-1 (±anti-CTLA-4) exposure; refractory/progressive disease	Checkpoint-refractory patients can still harbor expandable tumor-reactive populations and may be suitable candidates	Clinical association; consistent across contemporary cohorts	Routine (history)	[29,58,66,186,199]
Tumor dependent	Tumor mutational burden (TMB)/neoantigen load	WES/panel-based TMB; neoantigen prediction pipelines (research); subtype context (acral/mucosal)	Higher antigenicity may support broader tumor reactivity; lower TMB may limit diversity/strength of responses	Translational + clinical association; not yet a validated selection threshold	Moderate (needs sequencing; more feasible with clinical panels)	[17,18,204,235]
Tumor dependent	Antigen presentation integrity (HLA class I; B2M; IFN pathway competence)	Genomics/IHC for HLA/B2M; transcriptional IFN programs	Defects can underlie primary/acquired resistance to ICI and may impair TIL recognition/effector function	Strong biologic rationale; translational evidence; prospective validation needed	Moderate–specialized (assay-dependent)	[13,22,24,84,236]
Tumor dependent	Baseline immune-inflamed phenotype (CD8 density; T-cell-inflamed/IFN-γ signatures)	CD8 IHC (intratumoral/invasive margin); gene-expression immune signatures	Inflamed tumors and higher CD8/IFN-γ programs appear more favorable for response	Clinical + translational association; reproducibility depends on assay and spatial definition	Moderate (IHC routine; signatures specialized)	[14,29,36,58,60,66,67,84,111,116]
Tumor dependent	Immune-excluded/desert patterns and suppressive myeloid networks	Spatial IHC/digital pathology; cell–cell neighborhood metrics (CD8–myeloid)	Exclusion/desert and unfavorable CD8–myeloid organization associated with lower response rates	Emerging spatial biomarker evidence in TIL cohorts	Specialized (digital pathology/spatial analytics)	[14,66,237,238,239]
Tumor dependent	Resistance programs (Wnt/β-catenin, TGF-β, stromal/angiogenic barriers)	Tumor transcriptomics; pathway signatures; spatial multi-omics	Associated with reduced immune infiltration/effector function and reduced response in translational studies	Translational evidence; clinical utility not yet established	Specialized (research-grade assays)	[14,40,84,204,240,241,242]
TIL product dependent	Total infused TIL dose and in vitro tumor reactivity	Infused cell count; functional assays (e.g., IFN-γ release/cytotoxicity against autologous tumor)	Higher dose and stronger in vitro reactivity correlate with higher ORR and longer PFS	Clinical association from early and contemporary cohorts	Moderate (cell counts routine; functional assays variable by platform)	[158,166]
TIL product dependent	CD8 enrichment and breadth of tumor recognition (multi-antigen reactivity; fewer bystanders)	Flow cytometry (CD8/CD4); functional breadth assays; phenotyping for bystander-like profiles	Enrichment for tumor-reactive CD8 and broad recognition favors efficacy; bystander predominance may dilute potency	Translational + clinical association; product-dependent	Specialized (requires product immunophenotyping/functional testing)	[19,28,29,58,166,185,186,194,213]
TIL product dependent	Tumor-reactive PD-1^+^ CD39^+^ CD8^+^ subset	Flow cytometry for PD-1/CD39; subset quantification	Enrichment indicates tumor-reactive populations and associates with improved outcomes	Translational evidence with clinical correlations	Specialized (product flow cytometry)	[29,58,60,154,175,233,242,244,245,246]
TIL product dependent	Differentiation state (less differentiated/TCF1^+^ progenitor-exhausted vs. terminal exhaustion)	Phenotyping (TCF1, exhaustion markers); differentiation profiling	Less differentiated/TCF1^+^ states associate with better persistence and durability; terminal exhaustion associates with poorer durability	Growing translational + clinical association; not yet standardized for release criteria	Specialized (flow/omics depending on marker set)	[16,29,58,60,129,165,166,178,200,213,244,247,248,261]
TIL product dependent	TCR clonotype architecture and persistence (tumor overlap; long-term persistence post-infusion)	TCR sequencing pre/post; overlap with baseline tumor clonotypes	Responders often receive tumor-overlapping clonotypes that persist long-term and may mediate durable control	Translational evidence with clinical correlations	Specialized (sequencing infrastructure; turnaround considerations)	[21,58,60,66,233,249]
Spatial organization/TME dependent	High CD8 density and favorable intratumoral/invasive-margin localization; permissive stroma/vasculature	Quantitative pathology (H&E/IHC); digital pathology; spatial density/proximity metrics	Higher CD8 density and favorable localization associated with improved outcomes and a permissive architecture	Clinical association (quantitative pathology) with emerging spatial refinement	Moderate (IHC routine; digital quantification varies by center)	[42,43,100,250,251,252,253,254]
Spatial organization/TME dependent	TLSs and B-cell-rich aggregates	H&E/IHC; spatial mapping of B cells, DCs, and T cells; TLS scoring	TLSs indicate organized local antigen presentation and are associated with improved prognosis and higher response rates to immunotherapy	Strong association in melanoma immunotherapy literature; relevance to TIL harvest/response supported	Moderate (pathology/IHC feasible; standardized scoring evolving)	[36,37,114,115,116,133,135,255]
Spatial organization/TME dependent	CD8–myeloid cell network states (incl. type I IFN-activated myeloid programs) and tumor-intrinsic spatial contexts	Spatial multi-omics; multiplex IHC; neighborhood analyses	Responders show favorable CD8–myeloid patterns and tumor-intrinsic differences even at similar bulk TIL densities	Emerging spatial multi-omics evidence in checkpoint-refractory TIL-treated cohorts	Specialized (research-grade assays)	[14,66,67,176,256,257,258]

Abbreviations: DCs, dendritic cells; ECOG, Eastern Cooperative Oncology Group; H&E, hematoxylin and eosin; HLA, human leukocyte antigen; IFN-γ, interferon gamma; IHC, immunohistochemistry; LDH, lactate dehydrogenase; ORR, overall response rate; PFS, progression-free survival; PS, performance status; TCR, T-cell receptor; TIL, tumor-infiltrating lymphocyte; TLSs, tertiary lymphoid structures; TMB, tumor mutational burden; TME, tumor microenvironment; WES, whole-exome sequencing; IL-2, interleukin 2; CBC, complete blood count; ICI, immune checkpoint inhibitor; PD-1, programmed cell death protein 1; CTLA-4, cytotoxic T-lymphocyte associated protein 4; B2M, beta-2 microglobulin; CD8, cluster of differentiation 8; Wnt, Wingless-related integration site; TGF, transforming growth factor; CD4, cluster of differentiation 4; CD39, cluster of differentiation 39; TCF1, T cell factor 1.

### 8.6. Treatment Algorithms and Positioning of TIL Therapy

When adding TIL therapy to the melanoma treatment plan, it is important to consider current standards and the specific needs of each patient and tumor. For most patients with unresectable or metastatic melanoma, ICIs, either anti-PD-1 alone or in combination, are still the first choice for systemic therapy. BRAF/MEK inhibitors are used for patients with BRAF-mutant disease who need quick disease control [2,196,208,217,222,262].

For patients whose disease progresses after anti-PD-1-based therapy, TIL-ACT is now seen as a possible salvage treatment for those who are good candidates. Studies show that TIL therapy can lead to better response rates than ipilimumab, ipilimumab plus nivolumab, or chemotherapy in this situation [11,12,174]. A team approach should be used at or soon after ICI failure to check if there is a resectable metastatic lesion for TIL harvest, to see if the patient can handle lymphodepletion and IL-2, and to plan manufacturing timelines [196,208,209,262]. In practice, TIL-ACT is considered for patients who have already tried at least one anti-PD-1-based therapy, are healthy enough for lymphodepleting chemotherapy and IL-2, and have at least one resectable lesion that could provide a viable TIL product [12,185,262].

For patients with BRAF-mutant disease, the order of using BRAF/MEK inhibitors is tailored to each person based on how fast the disease is progressing, symptoms, and past treatments. TIL-ACT is usually considered after targeted therapy has failed or between rounds of ICI and targeted therapy in selected patients [196,197,222,225,262,263,264,265,266,267]. As new biomarkers are developed, these treatment plans may change to include factors like tumor inflammation, antigen presentation, and the expected quality of the TIL product. Future studies that compare different treatment orders and combinations, such as giving ICIs before TIL-ACT or combining TIL-ACT with ongoing checkpoint blockade, will help determine the best way to use TIL therapy in personalized care.

## 9. Strategies to Enhance TIL Therapy and Overcome Resistance

### 9.1. Engineering and Selecting Improved TIL Products

One way to improve TIL therapy is by refining the product through selective enrichment and genetic engineering. Standard products have polyclonal tumor-reactive T cells, but their effectiveness can be limited by exhaustion, sensitivity to inhibitory cytokines, and the need for high-dose IL-2 [157,158,166,197,268,269,270]. Enriching for PD-1^+^ or CD39^+^ CD8^+^ T cells from tumor samples increases the number of neoantigen-specific, tumor-resident clonotypes in the final product and has been linked to better functional potency [29,58,66,175,244,245]. Manufacturing methods that support less differentiated, TCF1^+^ progenitor-exhausted or central memory-like phenotypes aim to boost proliferative capacity and long-term persistence after infusion [129,130,157,165,178,271,272,273,274,275].

Genetic engineering methods are used to protect TILs from strong immunosuppressive signals. Knocking out PD-1 or blocking its signaling with CRISPR-based tools increases effector function and cytokine production in preclinical studies [276,277,278,279,280,281,282]. Making TILs resistant to TGF-β, for example by using dominant-negative TGF-β receptors, can protect them from suppression by the tumor environment and improve their antitumor effects [283,284,285,286,287,288,289]. TILs engineered to express cytokines like IL-12 show stronger local immune responses, but they can also cause systemic toxicity, highlighting the need for careful control of these systems [207,290,291,292,293]. In addition to these changes, optimizing culture conditions with other cytokines (such as IL-7 and IL-15), adjusting TCR stimulation, or adding CD40L support and B-cell-rich co-cultures can help expand TILs from tumors with few immune cells while keeping them less differentiated [157,232,294,295,296,297,298,299,300,301]. All these strategies aim to create TIL products that are more specific to tumors, last longer, and resist suppression.

### 9.2. Modifying the Tumor Microenvironment

The tumor microenvironment poses several challenges to TIL activity, including stromal exclusion, suppressive myeloid cells, abnormal blood vessels, and metabolic stress. As discussed in Section 3.7, when Wnt/β-catenin, TGF-β, VEGF, and MAPK pathways are active in tumors, they reduce dendritic-cell recruitment, lower antigen presentation, and limit lymphocyte movement, which leads to immune exclusion. These factors, along with epithelial–mesenchymal transition programs, have been linked to poor responses to TIL-ACT in translational studies [14,40,41,92,204,240,302,303,304,305]. Researchers are now testing inhibitors of TGF-β, VEGF, and related pathways to remodel the stroma and blood vessels, aiming to make tumors more accessible to TILs.

Myeloid cells and metabolic issues present more chances for intervention. Tumor-associated macrophages and myeloid-derived suppressor cells can limit T cell activity by releasing IL-10, TGF-β, arginase-1, and nitric oxide, and are often found in immune-excluded or immune-desert tumors [74,76,80,122,306,307,308,309,310]. Drugs that deplete or reprogram these cells, like CSF1R or PI3Kγ inhibitors, may boost TIL function. STING or toll-like receptor agonists can also activate dendritic cells and improve cross-presentation [70,311,312,313,314,315,316,317]. Other strategies, such as improving T-cell metabolic fitness or blocking immunosuppressive metabolites like adenosine and lactate, help restore effector function in preclinical models [53,54,318,319,320,321,322,323,324,325]. Together, these approaches aim to create a microenvironment that better supports the movement, survival, and activity of infused TILs.

### 9.3. Combination Strategies

Combining TIL therapy with other treatments can help target different mechanisms of action. ICIs are a logical choice, since checkpoint blockade can broaden and boost the body’s own T-cell responses, while TIL-ACT supplies many tumor-reactive cells. Research shows that blocking PD-1 can improve the function and survival of transferred T cells, especially those that are progenitor-exhausted or tumor-resident clonotypes [13,30,129,146,272,326,327,328,329,330]. In practice, most patients who get TIL-ACT have already received ICIs, and TIL cultures often include tumor-resident cells influenced by earlier checkpoint therapy [29,58,174,196,206]. Early-phase trials are now testing anti-PD-1 given at the same time or after TIL-ACT to keep the treatment working longer and lower the chance of relapse [262,331,332].

For BRAF-mutant melanoma, targeted therapy is another possible solution. BRAF/MEK inhibitors can temporarily raise melanoma antigen levels, lower immunosuppressive cytokines, and help T cells enter tumors, which may improve the quality of TILs collected for expansion [87,333,334,335,336,337,338,339]. However, these drugs also change the tumor’s clonal structure and can cause resistance, which may limit long-term results. The best way to sequence these treatments with TIL-ACT is still unclear [196,225,262,263,266,340]. Other local or intratumoral treatments, like radiotherapy, oncolytic viruses, and TLR or STING agonists, can trigger immunogenic cell death, increase antigen release, and boost dendritic-cell activation and chemokine production. This can make the tumor environment more supportive for infused T cells [70,341,342,343,344,345,346,347,348,349,350]. Most of these combinations are still being studied, and it is important to watch for toxicity, timing, and possible negative interactions. Still, combining ICIs, targeted drugs, and therapies that change the tumor environment could help more patients benefit from TIL-ACT.

### 9.4. Innovations in Manufacturing and Logistics

Recent progress in TIL manufacturing and logistics is important for making these therapies more widely available. Centralized platforms, like those used for lifileucel, use standardized and partly automated workflows with set culture conditions, quality controls, and proven cryopreservation methods. This helps ensure consistent production of clinical-grade TIL products at different sites and from various tumor samples [11,157,187,206,207,262,351,352,353]. One main goal is to shorten the time from cell collection to infusion, which is especially important for patients whose disease is progressing quickly. Improvements such as starting pre-REP cultures earlier, better selection of viable tissue fragments, faster REP protocols, and more efficient release testing are all aimed at speeding up manufacturing while keeping product quality high [184,193,194,352,354,355,356]. Storing intermediate TIL banks by cryopreservation also gives more flexibility in scheduling lymphodepletion and infusion, so there is less reliance on finishing cultures in real time [11,186,197,209,243,262].

Automation and closed-system bioreactors are designed to reduce manual handling, lower the risk of contamination, and cut down on labor, which helps meet good manufacturing standards. These advances could also make it possible to produce TILs at regional centers or hospitals, though current systems still need significant investment and technical skill [157,193,357,358,359]. Academic centers are working to add new selection or activation steps, like PD-1^+^ or CD39^+^ enrichment, into processes that can be scaled up without losing reliability [167,180,181,360,361]. In addition to these technical improvements, efforts are underway to standardize tissue shipping, centralize scheduling, and align clinical pathways to support referral networks and make TIL therapy more accessible [197,262,362]. All of these changes are important for moving TIL-ACT from a specialized procedure to a treatment that can be used more widely in routine care (Table 4).

## 10. Challenges and Future Directions

### 10.1. Practical and Biological Limitations of TIL Therapy

Although TIL therapy has shown it can work, there are still major practical and biological challenges. Practically, TIL-ACT is demanding. It needs surgery to collect tumor tissue, specialized production under strict standards, and long hospital stays for chemotherapy and high-dose IL-2, all managed by experienced oncology teams [160,196,262,372]. These steps mean only certain centers can offer the treatment, making it usually out of reach for patients in places with fewer resources. Sometimes, tumors do not provide enough or good-quality TILs, especially if there are few T cells to start with or a lot of tissue damage. Also, the time needed for therapy production may not fit with a fast-growing disease [11,161,166,188,193,208,209,243,262,352,356,373]. The cost of making these personalized cells and the hospital care is high, and it is still unclear if this approach is cost-effective compared to other advanced treatments.

Biologically, not all patients respond the same way to TIL-ACT. Many do not benefit or may relapse even after receiving tumor-reactive T cells [11,12,14,66,160,174,374]. Tumors can resist treatment through mechanisms like poor antigen presentation, loss of antigens, or activation of certain pathways such as Wnt/β-catenin, TGF-β, or VEGF. Other factors, like suppressive immune cells, metabolic stress, and tough tumor environments, also lower the efficacy of TIL therapy [14,22,40,41,74,91,204,363,364,365,366,367,368,369,370,371]. There are no reliable biomarkers yet to predict which patients will benefit most. While some features (like T cell-inflamed tumors, good antigen presentation, helpful tumor structures, and certain T cell types) are linked to better responses, these are not yet used regularly in clinical decisions [14,29,36,58,66,175,199,233,238,242,262,375,376,377]. Addressing these practical, financial, and biological challenges is key to making TIL therapy more widely available.

### 10.2. Key Research Priorities and Future Directions

Key research priorities are emerging for advancing TIL therapy in melanoma. One important need is to validate integrated biomarkers that combine clinical factors, tumor immune context, spatial features, and TIL product characteristics to help guide patient selection and timing. Factors like T cell-inflamed gene signatures, antigen presentation status, CD8^+^ T cell–myeloid cell networks, and the dominance of tumor-resident clonotypes have been strongly linked to outcomes in translational studies. However, these need to be standardized and included in prospective trials before they can be used in routine decisions [14,29,36,58,60,66,209,233,238,242,262,375,376,378].

Improving TIL product design is another important focus. New strategies are being tested to enrich for neoantigen-specific and less differentiated T-cell subsets, adjust exhaustion programs, and make T cells more resistant to inhibitory pathways like PD-1 and TGF-β. These approaches are in early clinical trials and may improve response rates and durability, especially when combined with better culture methods and scalable manufacturing [60,153,165,276,278,290,352,379,380,381,382]. It is also important to systematically test combination treatments. Trials are evaluating TIL-ACT with checkpoint inhibitors, targeted therapy, radiotherapy, or intratumoral immunomodulators to overcome resistance in the tumor environment and help patients with immune-excluded or low-TMB tumors [332,333,342].

Research on manufacturing, health economics, and implementation science is crucial to make TIL therapy more widely available. Efforts to speed up the process from collection to infusion, lower costs through automation and standard platforms, and adjust workflows for different healthcare systems are important steps to move TIL-ACT from a specialized procedure to a regular part of melanoma care [11,193,208,209,262,352,383]. In the future, trials based on biomarkers, next-generation TIL products with better specificity and persistence, and research focused on cost and access are likely to have the biggest impact on clinical practice. These advances will help establish TIL-ACT as a personalized treatment for some patients with advanced melanoma and may support expanding TIL-based approaches to other solid tumors.

## 11. Conclusions

Melanoma is a cancer that strongly interacts with the immune system, and TILs act as both markers and treatments. Patients usually do better and respond more to ICIs when TILs contain a large number of CD8^+^ T cells, continue to present antigens, and are found in environments with active T cells. Recent studies using single-cell and spatial profiling have found that tumor-resident clonotypes, connections between CD8^+^ T cells and myeloid cells, and TLSs are all important for local immune control.

Adoptive cell therapy with TILs grown outside the body has moved from early experiments to a standard treatment that can lead to strong and lasting responses in people with advanced melanoma, even when other immune therapies have not worked. Current evidence, including studies with lifileucel and comparisons with ipilimumab, shows response rates of about 30–50% and long-term remissions in some patients with few other options. These findings show that TIL-ACT can boost antitumor immunity by increasing the number of tumor-resident, neoantigen-specific T cells.

Still, TIL therapy has challenges, including complex logistics and treatment-related side effects, as well as resistance due to antigen loss, issues with IFN signaling, and immune-excluded environments. To move forward, progress will depend on better patient selection, smart combinations that alter the tumor microenvironment, and new TIL products that target more effectively, last longer, and resist suppression.

In summary, current evidence indicates that TIL-based therapy is an important component of personalized treatment for advanced melanoma, particularly after PD-1 therapy. Ongoing research will help improve its use, make it easier to access, and guide its use in other solid tumors.

## Figures and Tables

**Table 2:** Key clinical trials of TIL adoptive cell therapy in advanced melanoma.

Trial	Design	Sample Size	Population/Prior Therapies	Conditioning	IL-2 Regimen	Outcomes (ORR/CR; DOR; PFS/OS)	Key Toxicity	References
NCI/NIH (TIL-ACT with escalating lymphodepletion ± TBI)	Single-center; sequential phase II cohorts	93	Metastatic melanoma; refractory to standard therapies (incl. prior IL-2/chemo)	Cy/Flu (nonmyeloablative); ± TBI (2 Gy or 12 Gy; 12 Gy with CD34^+^ rescue)	High-dose bolus IL-2	ORR: 49% (NMA regimen), 52% (2 Gy), 72% (12 Gy) CR: 12%, 20%, 40% Durable CRs reported OS: 3-yr 36%; 5-yr 29%	Severe cytopenias and infectious risk; IL-2–related toxicities; 1 treatment-related death	[158,160,200]
MD Anderson (expanded autologous TIL; “young TIL” approach)	Single-center phase II (ongoing)	31	Metastatic melanoma unresponsive to conventional therapies	Transient lymphodepletion (chemotherapy; per protocol)	Two cycles high-dose IL-2	ORR: 48.4% CR: 6.5% PFS: >12 mo in 9/15 responders (60% of responders) OS: NR	Expected lymphodepletion and IL-2 toxicities (hospital-based supportive care)	[166]
Denmark (Herlev/CCIT): TIL-ACT with attenuated IL-2	Single-center phase I/II	25	Progressive, treatment-refractory metastatic melanoma; age < 70; good performance; ≥1 resectable metastasis	Standard lymphodepleting chemotherapy	Attenuated IV continuous “decrescendo” IL-2	ORR: 42% (CR 3; PR 7) DOR: long-lasting CRs reported OS: median 21.8 mo PFS: NR	IL-2 toxicities observed but manageable on oncology ward without ICU	[9]
Lifileucel (LN-144), C-144-01 (initial report; cohort 2)	Multicenter, single-arm phase II	66	Advanced melanoma; heavily pretreated (mean 3.3 prior lines); 100% anti-PD-1, 80% anti-CTLA-4; 23% BRAF/MEK (if BRAF-mutant)	Nonmyeloablative lymphodepleting chemotherapy	Up to 6 doses high-dose IL-2	ORR: 36% (CR 2; PR 22) DOR: median NR (median FU 18.7 mo) PFS/OS: NR (initial report)	Safety profile consistent with lymphodepletion + IL-2	[11]
Lifileucel, C-144-01 pooled analysis (consecutive cohorts)	Multicenter pooled analysis (single-arm)	153	Advanced melanoma; median 3 prior lines; 81.7% prior anti-PD-1 and anti-CTLA-4	Nonmyeloablative lymphodepleting chemotherapy	Up to 6 doses high-dose IL-2	ORR: 31.4% (CR 5.2%) DOR: median NR (median FU 27.6 mo) PFS: median 4.1 mo OS: median 13.9 mo	Most common grade 3/4 TRAEs: thrombocytopenia (76.9%), anemia (50.0%), febrile neutropenia (41.7%)	[174]
Randomized phase III (TIL vs. ipilimumab; TIL-IMP/NCT02278887)	Multicenter, open-label phase III RCT (1:1)	168 (84/arm)	Unresectable stage III/IV melanoma; 86% anti-PD-1-refractory; prior systemic therapy (mostly adjuvant or first-line anti-PD-1)	Cy/Flu (nonmyeloablative); TIL infusion ≥ 5 × 10^9^ cells (median 40.9 × 10^9^ in treated pts)	High-dose IL-2 (median 4 doses; range 0–10 in treated pts)	ORR: 49% vs. 21% CR: 20% vs. 7% PFS: 7.2 vs. 3.1 mo (HR 0.50) OS: 25.8 vs. 18.9 mo	Grade ≥ 3 TRAEs: 100% (TIL) vs. 57% (ipilimumab); mainly chemotherapy-related myelosuppression in TIL arm	[12]

Abbreviations: ACT, adoptive cell therapy; CR, complete response; CTLA-4, cytotoxic T-lymphocyte-associated protein 4; Cy, cyclophosphamide; DOR, duration of response; Flu, fludarabine; FU, follow-up; HR, hazard ratio; ICU, intensive care unit; IL-2, interleukin-2; IV, intravenous; mo, months; NMA, nonmyeloablative; NR, not reached/not reported; ORR, overall response rate; OS, overall survival; PD-1, programmed cell death protein 1; PFS, progression-free survival; PR, partial response; pts, patients; RCT, randomized controlled trial; TBI, total-body irradiation; TIL, tumor-infiltrating lymphocytes; TRAEs, treatment-related adverse events; NCI, National Cancer Institute; NIH, National Institutes of Health; Gy, Gray; CD34, Cluster of differentiation 34; CCIT, Centre for Clinical Investigation and Therapeutics; LN-144, lifileucel; BRAF, V-Raf Murine Sarcoma Viral Oncogene Homolog B; MEK, Mitogen-activated extracellular signal-regulated kinase; IMP, Investigational Medicinal Product; NCT, National Clinical Trial.

**Table 4:** Strategies to enhance TIL efficacy and reduce toxicity.

Intervention	Rationale	Representative Trial Directions/Examples	References
Conditioning regimen optimization (avoid TBI; tailored lymphodepletion intensity)	Maintain the cytokine-rich niche and deplete suppressive/competing lymphocytes while limiting hematologic and infectious toxicity.	Comparative studies of reduced-intensity or alternative conditioning; individualized regimens for older/comorbid patients.	[9,158,159,160,161,165,186,198,199,200]
Cytokine support optimization (IL-2 dose-capping/reduced courses; IL-2 variants and alternative γ-chain cytokines)	Support engraftment and expansion of infused TILs while reducing capillary leak, organ dysfunction, and need for ICU-level care.	Fixed maximum IL-2 dosing (e.g., up to 6 doses), intermediate/low-dose schedules; evaluation of IL-2 variants or cytokines such as IL-7/IL-15 to enhance persistence with lower toxicity.	[9,11,15,160,166,186,196,201,202,203,204,205,206]
Stringent eligibility criteria, pre-treatment workup, and standardized supportive care pathways	Reduce treatment-related morbidity and enable patients to complete lymphodepletion and cytokine support safely.	Consensus-driven selection (ECOG 0–1; cardiac/pulmonary/renal reserve), infection screening, transfusion and antimicrobial prophylaxis pathways, and post-infusion monitoring protocols.	[160,186,196,197,207,208]
Enrichment for tumor-reactive T cells (e.g., PD-1^+^ or CD39^+^ selection)	Increase product potency by reducing bystander lymphocytes and enriching for tumor-reactive clonotypes.	Incorporate selection/enrichment into GMP-compatible workflows; combine with functional reactivity readouts to prioritize highly reactive fragments.	[29,58,66,157,158,166,167,175,180,181,197,244,245,268,269,270,360,361]
Manufacturing to preserve stem-like/less differentiated states (shorter culture; limit terminal differentiation)	Stem-like/progenitor-exhausted T cells exhibit superior proliferative capacity and persistence after transfer, supporting durable responses.	Shortened culture/accelerated REP strategies; culture conditions that retain TCF1^+^ and memory-like phenotypes.	[129,130,157,165,178,271,272,273,274,275]
PD-1 pathway disruption in TILs (e.g., CRISPR editing)	Reduce inhibitory checkpoint signaling within the tumor microenvironment and sustain effector function.	Engineered/edited TIL products evaluated in early-phase clinical studies; ongoing optimization of editing efficiency and safety.	[276,277,278,279,280,281,282]
Engineering resistance to suppressive cytokines (dominant-negative TGF-β receptor)	Enable transferred TILs to function in TGF-β-rich, immune-excluded melanoma microenvironments.	Clinical translation of TGF-β-resistant engineered TILs; combinations with stromal/vascular remodeling approaches.	[283,284,285,286,287,288,289]
Cytokine-armored TILs (e.g., IL-12 expression)	Augment local inflammation, antigen presentation, and effector function to overcome suppression and improve tumor control.	Inducible or regulated IL-12 “armored” TIL constructs to balance potency and safety; exploration in solid-tumor ACT programs.	[207,290,291,292,293]
Ex vivo culture enhancements (alternative cytokines; modified stimulation; CD40L/B-cell-rich co-cultures)	Improve expansion of functional tumor-reactive T cells, reduce exhaustion, and support CD4/CD8 cooperation.	Use of IL-7/IL-15-based culture, modified activation, and APC-supportive co-cultures; adaptation to scalable GMP platforms.	[157,232,294,295,296,297,298,299,300,301]
Tumor-intrinsic barrier targeting (immune exclusion pathways such as Wnt/β-catenin; antigen presentation defects)	Reverse immune-excluded/immune-desert phenotypes and improve trafficking/recognition of infused TILs.	Combination strategies incorporating pathway modulation and stroma/vasculature remodeling (e.g., TGF-β/VEGF-axis inhibition) to render tumors permissive to infiltration.	[14,22,40,41,91,92,204,240,302,303,304,305,363,364,365,366,367,368,369,370,371]
Myeloid reprogramming and innate activation (CSF1R/PI3Kγ inhibition; STING/TLR agonists)	Decrease suppressive macrophage/MDSC activity and enhance dendritic-cell priming and cross-presentation.	Combinations of TIL-ACT with myeloid-targeting agents; systemic or intratumoral innate agonists to boost antigen presentation and chemokine production.	[70,74,76,80,122,306,307,308,309,310,311,312,313,314,315,316,317]
Metabolic interventions (adenosine/lactate axis; improving T-cell metabolic fitness)	Overcome metabolic stress and inhibitory metabolites that blunt T-cell effector function in the tumor microenvironment.	Pair TIL-ACT with agents targeting adenosine signaling or lactate/acidic stress; integrate metabolic fitness readouts in product characterization.	[53,54,318,319,320,321,322,323,324,325]
Checkpoint inhibitor combinations (concurrent or sequential anti-PD-1; post-infusion maintenance)	Enhance persistence and function of transferred T cells and broaden endogenous immunity; reduce relapse after infusion.	Trials evaluating concurrent/sequential anti-PD-1 with TIL-ACT; maintenance checkpoint blockade strategies post-infusion.	[13,30,129,146,262,272,326,327,328,329,330,331,332]
Targeted therapy combinations in BRAF-mutant melanoma (BRAF/MEK inhibitors)	Transiently increase antigen expression and T-cell infiltration and potentially improve the quality of harvested TILs.	Optimization of sequencing (pre-harvest priming vs. peri-infusion); trials defining benefit-risk and resistance interactions.	[87,196,225,262,263,266,333,334,335,336,337,338,339,340]
Locoregional/intratumoral immunomodulation (radiotherapy; oncolytic viruses; intratumoral TLR/STING agonists)	Induce immunogenic cell death and antigen release, enhance dendritic-cell activation, and promote chemokine-driven trafficking of infused T cells.	Integrate radiation or intratumoral agents as priming/bridging to infusion; evaluate synergy and toxicity in early-phase combinations.	[70,341,342,343,344,345,346,347,348,349,350]
Centralized and rapid manufacturing plus cryopreservation (shorten vein-to-vein time)	Increase feasibility and reproducibility, reduce manufacturing failure and delays that can lead to clinical deterioration before infusion.	Standardized centralized workflows; accelerated pre-REP/REP and streamlined release testing; cryopreserved intermediate banks to flex scheduling of conditioning/infusion.	[11,157,184,186,187,193,194,206,207,209,243,262,351,352,353,354,355,356]
Automation/closed-system bioreactors and scalable integration of selection/activation steps; coordinated referral pathways	Reduce contamination risk and labor burden, enable regional/hospital-based production, and facilitate timely access across networks.	Closed/semiautomated GMP protocols; integration of PD-1^+^/CD39^+^ enrichment; standardized tissue shipping and scheduling pathways.	[157,167,180,181,193,197,262,357,358,359,360,361,362]

Abbreviations: ACT, adoptive cell therapy; APC, antigen-presenting cell; BRAF, V-Raf Murine Sarcoma Viral Oncogene Homolog B; CSF1R, colony-stimulating factor 1 receptor; ECOG, Eastern Cooperative Oncology Group; GMP, good manufacturing practice; ICU, intensive care unit; IL, interleukin; MDSC, myeloid-derived suppressor cell; MEK, mitogen-activated extracellular signal-regulated kinase; PD-1, programmed cell death protein 1; PI3Kγ, phosphoinositide 3-kinase gamma; REP, rapid expansion protocol; STING, stimulator of interferon genes; TBI, total-body irradiation; TGF-β, transforming growth factor beta; TILs, tumor-infiltrating lymphocytes; TLR, toll-like receptor; VEGF, vascular endothelial growth factor; IL-2, interleukin 2; IL-15, interleukin 15; CD39, cluster of differentiation 39; TCF1, T cell factor 1; CRISPR, clustered regularly interspaced short palindromic repeats; IL-12, interleukin 12; CD40L, cluster of differentiation 40 ligand; CD4, cluster of differentiation 4; CD8, cluster of differentiation 8; IL-7, interleukin 7; Wnt, Wingless-related integration site.

## Data Availability

No new data were created or analyzed in this study. Data sharing is not applicable to this article.

## Abbreviations

The following abbreviations are used in this manuscript:

ACT	Adoptive cell therapy
APC	Antigen-presenting cell
BRAF	V-Raf Murine Sarcoma Viral Oncogene Homolog B
CD	Cluster of differentiation
cDC1	Conventional type 1 dendritic cells
CI	Confidence interval
CR	Complete response
CSF1R	Colony-stimulating factor 1 receptor
CTLA	Cytotoxic T-lymphocyte-associated protein
CXCL	C-X-C motif chemokine ligand
Cy	Cyclophosphamide
Cy/Flu	Cyclophosphamide/fludarabine
DC	Dendritic cell
DOR	Duration of response
ECOG	Eastern Cooperative Oncology Group
Flu	Fludarabine
FU	Follow-up
FOXP3	Forkhead box P3
GMP	Good manufacturing practice
gp100	Glycoprotein 100
HD	High-dose
HLA	Human leukocyte antigen
HPF	High-power field
HR	Hazard ratio
H&E	Hematoxylin and eosin
ICI	Immune checkpoint inhibitor
ICU	Intensive care unit
IFN	Interferon
IFN-γ	Interferon-gamma
IHC	Immunohistochemistry
IL	Interleukin
IV	Intravenous/intravenously
JAK	Janus kinase
LAG-3	Lymphocyte-activation gene 3
LDH	Lactate dehydrogenase
MAPK	Mitogen-activated protein kinase
MART-1	Melanoma-associated antigen recognized by T-cells 1
MDSC	Myeloid-derived suppressor cell
MEK	Mitogen-activated extracellular signal-regulated kinase
mo	Months
NCI	National Cancer Institute
NMA	Nonmyeloablative
NR	Not reached/not reported
NY-ESO-1	New York esophageal squamous cell carcinoma-1
ORR	Overall response rate
OS	Overall survival
PD-1	Programmed cell death protein
PFS	Progression-free survival
PI3Kγ	Phosphoinositide 3-kinase gamma
PR	Partial response
pre-REP	Pre-rapid expansion phase
PS	Performance status
pts	Patients
QC	Quality control
RCT	Randomized controlled trial
RECIST	Response Evaluation Criteria in Solid Tumors
REP	Rapid expansion protocol
ROI	Region of interest
SLN	Sentinel lymph node
STING	Stimulator of interferon genes
TBI	Total-body irradiation
TCF	T-cell factor
TCR	T-cell receptor
TGF-β	Transforming growth factor-beta
TIL	Tumor-infiltrating lymphocyte
TIL-ACT	TIL-based adoptive cell therapy
TLS	Tertiary lymphoid structure
TMB	Tumor mutational burden
TME	Tumor microenvironment
TOX	Thymocyte selection-associated high mobility group box
TRAE	Treatment-related adverse event
Treg	Regulatory T-cell
TRM	Tissue-resident memory
UV	Ultraviolet
VEGF	Vascular endothelial growth factor
WES	Whole-exome sequencing
